# Genome Sequence of Streptomyces cavourensis 1AS2a, a Rhizobacterium Isolated from the Brazilian Cerrado Biome

**DOI:** 10.1128/MRA.00065-19

**Published:** 2019-05-02

**Authors:** Harold Alexander Vargas Hoyos, Suikinai Nobre Santos, Gabriel Padilla, Itamar Soares Melo

**Affiliations:** a Laboratory of Environmental Microbiology, Embrapa Environment, Brazilian Agricultural Research Corporation–EMBRAPA, Jaguariúna, São Paulo, Brazil; b Biomedical Sciences Institute, University of São Paulo, São Paulo, São Paulo, Brazil; University of Arizona

## Abstract

Streptomyces cavourensis strain 1AS2a, isolated from wheat rhizosphere in the Brazilian Neotropical savanna, exhibits strong antimicrobial activities. Its genome comprises 7,600,475 bp with 6,590 open reading frames (ORFs) that reveal 30 biosynthetic gene clusters (BGCs). It provides a genetic basis for further research of the potential of this strain for the production of antimicrobial compounds.

## Abstract (precis)

Streptomyces cavourensis strain 1AS2a, isolated from wheat rhizosphere in the Brazilian Neotropical savanna, exhibits strong antimicrobial activities. Its genome comprises 7,600,475 bp with 6,590 open reading frames (ORFs) that reveal 30 biosynthetic gene clusters (BGCs).

## ANNOUNCEMENT

The Brazilian Neotropical savanna (Cerrado) covers more than 20% of Brazil and has been identified as one of the world’s biodiversity hotspots. However, although the biodiversity of this biome has not yet been thoroughly explored, recent efforts have highlighted the importance of *Actinobacteria* in the Cerrado (1). Recent studies have described *Actinobacteria* as important producers of compounds with agricultural applications, including antiparasitics, fungicides, larvicides, and nematicides (2). The genus Streptomyces is the largest and most prominent group of the phylum *Actinobacteria* with biological applications (3), and almost 1,000 species have been identified from different aquatic and terrestrial environments, mainly in soils and sediments (4).

Streptomyces cavourensis 1AS2a was isolated from a wheat crop in the Brazilian Cerrado, which is located in the middle-west region close to Brasilia DF (15°36′S, 47°42′W). Serial dilutions of the rhizospheric soil were inoculated on International *Streptomyces* Project 2 (ISP-2) medium at 30°C for 5 days; isolation and purification were made considering the morphological similarity of S. cavourensis 1AS2a with other Streptomyces species (5). Acidic-pH crude extract of *S. cavourensis* 1AS2a was obtained with ethyl acetate solvent (6) and exhibited antimicrobial *in vitro* activity against Sclerotinia sclerotiorum, Micrococcus luteus, Escherichia coli, and Pythium aphanidermatum.

Genomic DNA was extracted from a colony pool obtained from *S. cavourensis* 1AS2a that was grown for 3 days at 28°C in ISP-2 broth at 140 rpm using the UltraClean microbial DNA kit (Mo Bio, USA). A draft genome assembly was generated from *S. cavourensis* 1AS2a using paired-end long sequencing with PacBio RS II technology (7) and PacBio P6-C4 chemistry. The library was constructed using BluePippin size selection, with an average fragment of 20 kb (range, 10 to 35 kb). Sequencing was performed using single-molecule real-time (SMRT) cells (8) in an RS II sequencer (UW PacBio Sequencing Services, University of Washington, Seattle, WA). Default parameters were used for all software programs, unless otherwise specified. The raw reads were assembled using Hierarchical Genome Assembly Process (HGAP; version 2.1.1, PacBio data), yielding 7.6 Mb, which combined into 1 contig with 143.1× coverage.

The complete genome of *S. cavourensis* 1AS2a was annotated using Rapid Annotations using Subsystems Technology (RAST) (9, 10). The genome size was determined to be 7,600,475 bp, containing a predicted 6,590 open reading frames (ORFs) and 435 subsystems, with a G+C content of 72.1 mol%. The genome contained 156 genes predicted to encode proteins with functions related to stress responses, including cold and heat shock, osmotic, detoxification, and oxidative stress.

In order to identify the BGCs (11) of *S. cavourensis* 1AS2a, additional genome annotation was performed using antiSMASH version 4.2 (12), which identified 30 BGCs, 10 of which matched known clusters for ectoine (13), desferrioxamine B (14), SRO 15-2005 (15), Amfs (16), macrotetrolide (17), bafilomycin (18), SGR_PTMs (19), melanin (20), alkylresorcinol (21), and isorenieratene (22); these had 100% similarity and two clusters encoding griseobactin (23) and coelichelin (24) at >70%. The remaining 18 clusters were predicted to encode polyketide synthase (PKS) types II and III, thiopeptide/PKS1/nonribosomal peptide synthetase (thiopeptide/PKS1/Nrps) hybrid, bacteriocin, aryl polyene, butyrolactone, lantipeptide, thiopeptide, siderophore, and butyrolactone/ectoine hybrid (one of each) proteins, as well as Nrps and terpene (4 of each). The genome sequence information of *S. cavourensis* 1AS2a will facilitate further studies of this strain as a promising source of novel bioactive compound producers, particularly as natural compounds for agricultural application.

### Data availability.

Raw sequencing data sets have been registered in the NCBI SRA database under accession number SRR8446491. This whole-genome sequencing (WGS) project has been deposited at DDBJ/EMBL/GenBank under the accession number CP024957 and BioProject number PRJNA419149. The version described in this paper is version CP024957.1.
